# Supplementary figures and images for: The autophagy receptor NBR1 directs the clearance of photodamaged chloroplasts
Source: eLife. 2023 Apr 18;12:e86030. doi: 10.7554/eLife.86030 (PMC10156165; doi:10.7554/eLife.86030)

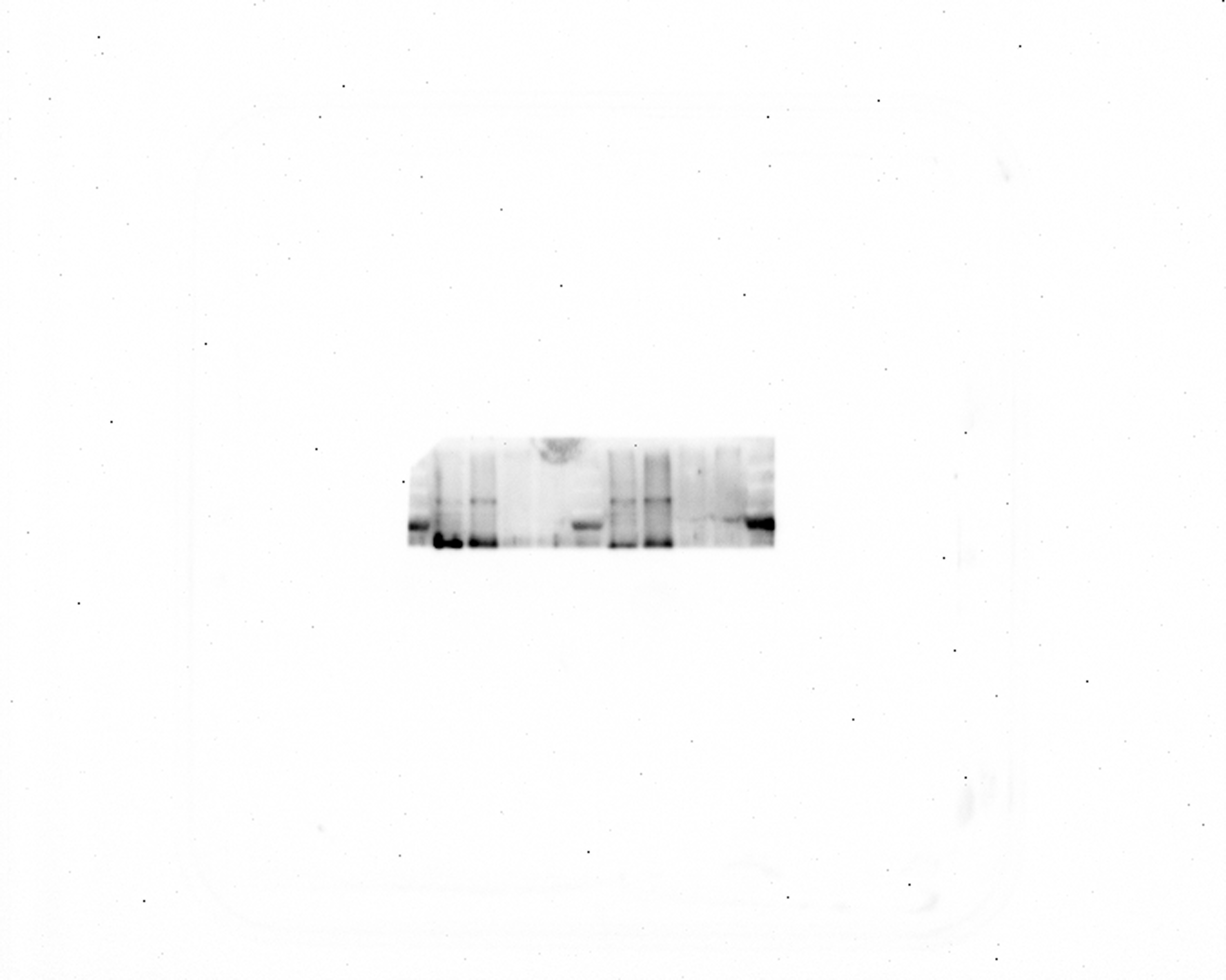

Supplement: Figure 2—source data 1. [file elife-86030-fig2-data1.zip › Figure 2G-source data/atg7-NBR1-CHEMI_04212022_160240_(Chemi).tif]

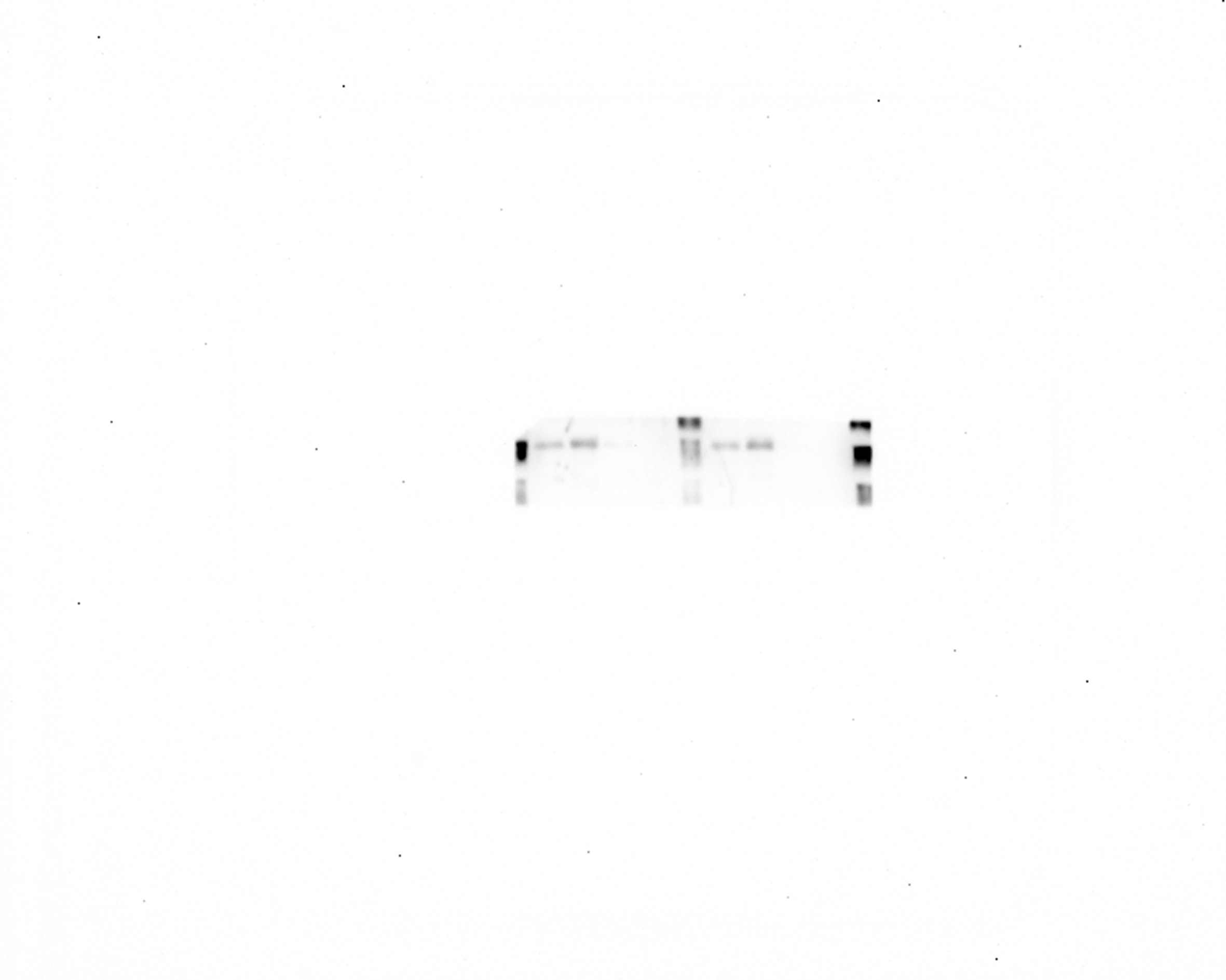

Supplement: Figure 2—source data 1. [file elife-86030-fig2-data1.zip › Figure 2G-source data/atg7-TIC40-CHEMI_04212022_161834_(Chemi).tif]

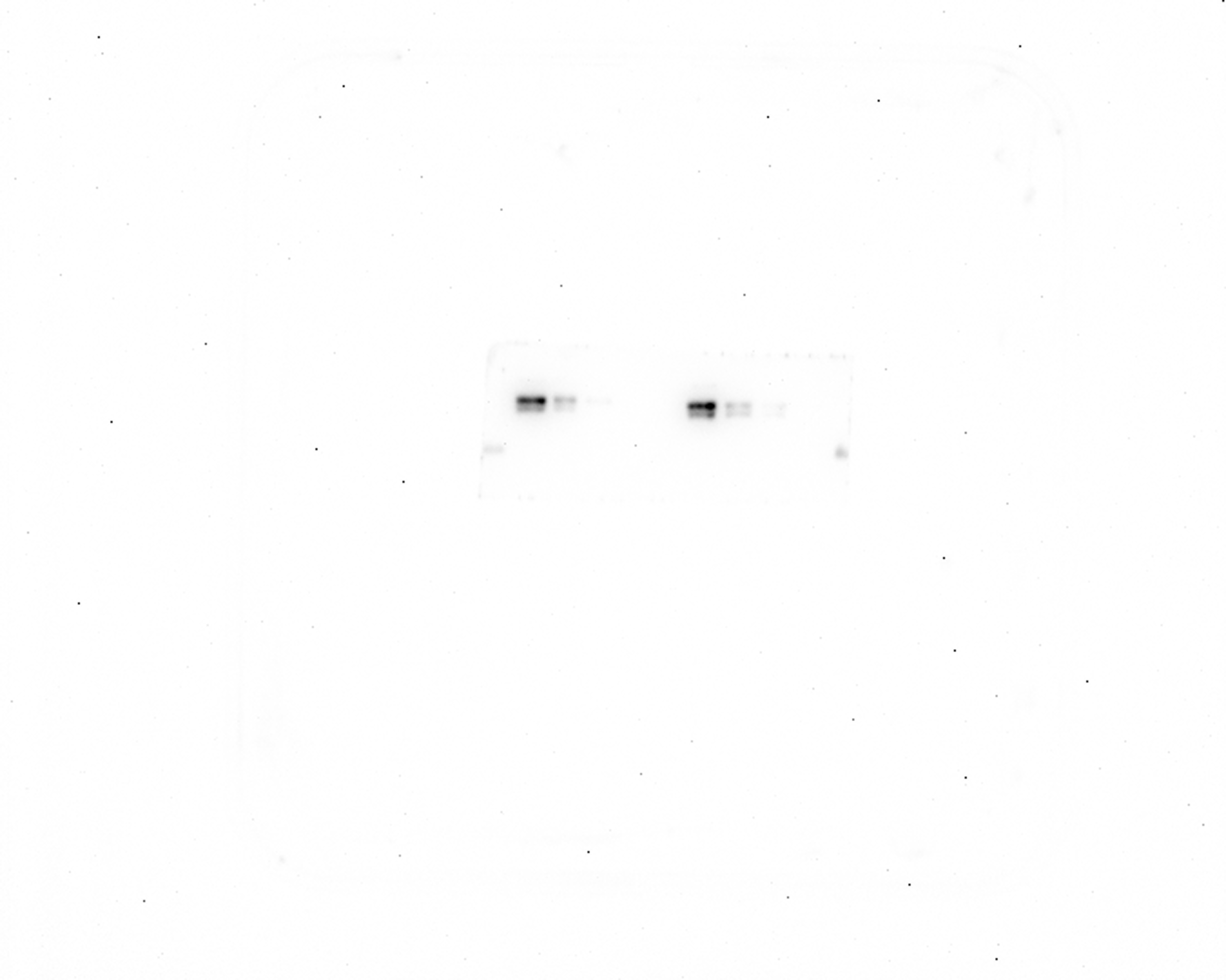

Supplement: Figure 2—source data 1. [file elife-86030-fig2-data1.zip › Figure 2G-source data/WT-cFBPAse-CHEMI_04212022_160913_(Chemi).tif]

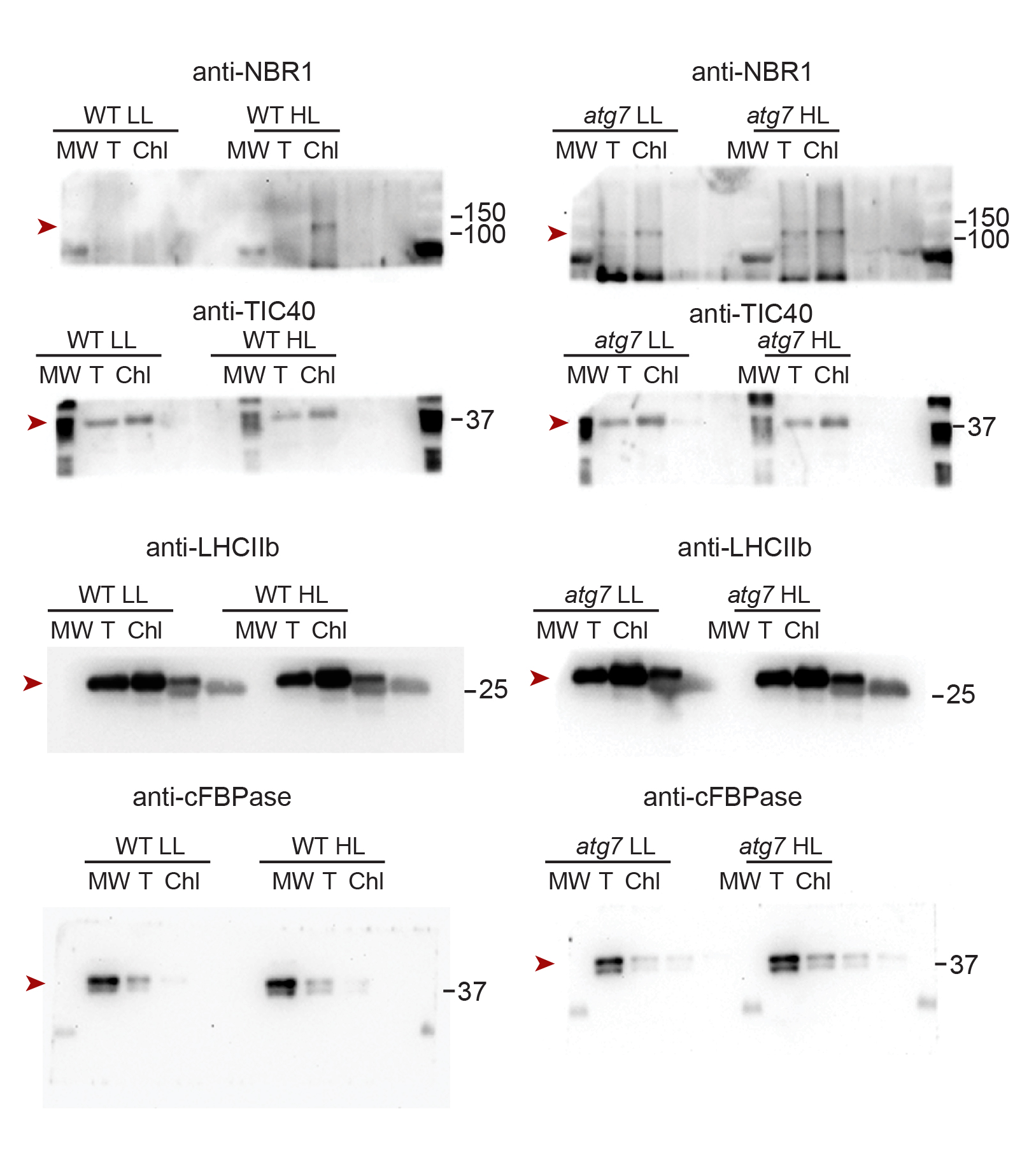


Uncropped immunoblots used in Figure 2G.

Supplement: Figure 2—source data 1. [file elife-86030-fig2-data1.zip › Figure 2G-source data/Uncropped immunoblots used in Figure 2G.docx]

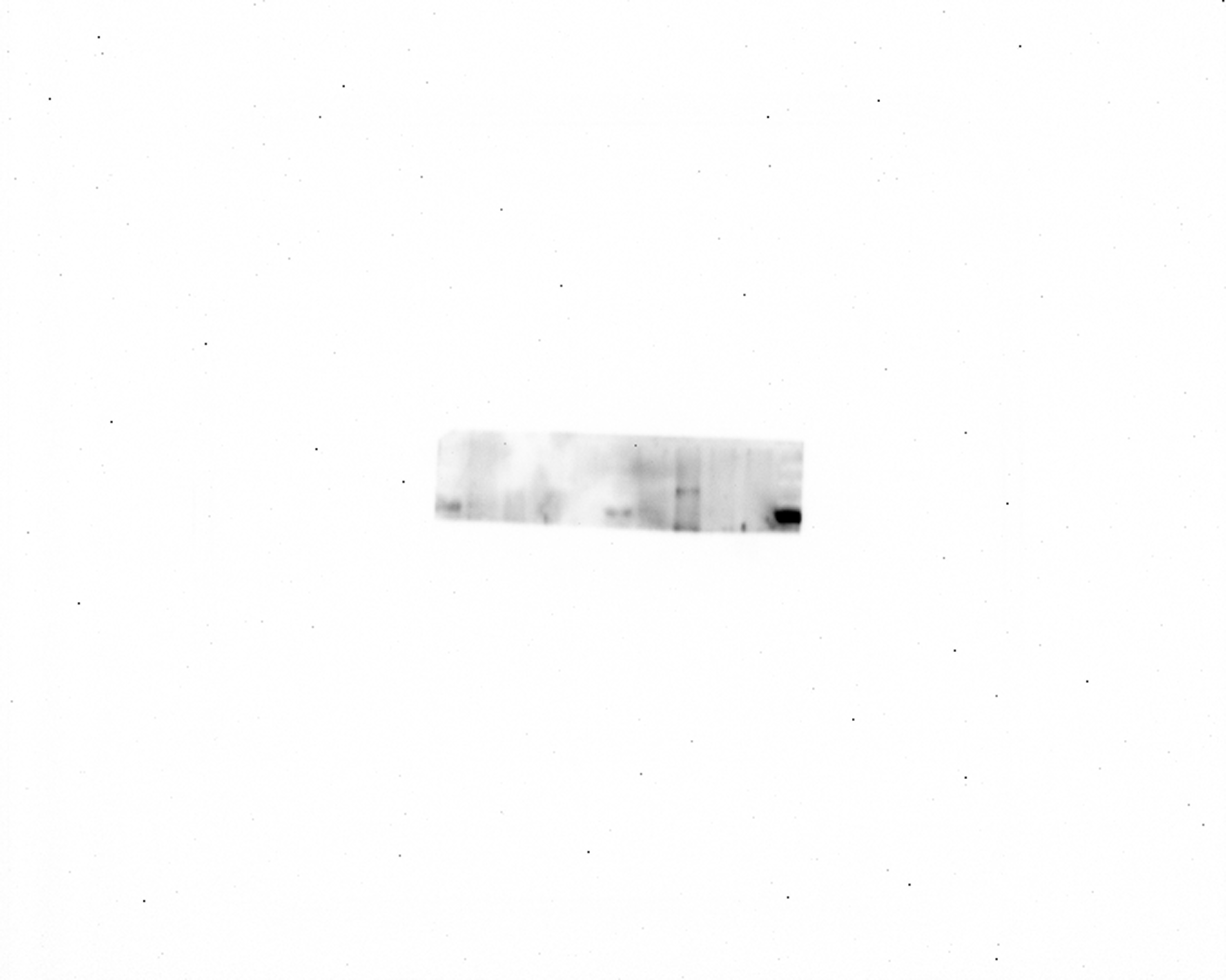

Supplement: Figure 2—source data 1. [file elife-86030-fig2-data1.zip › Figure 2G-source data/WT-NBR1-CHEMI_04212022_155807_(Chemi).tif]

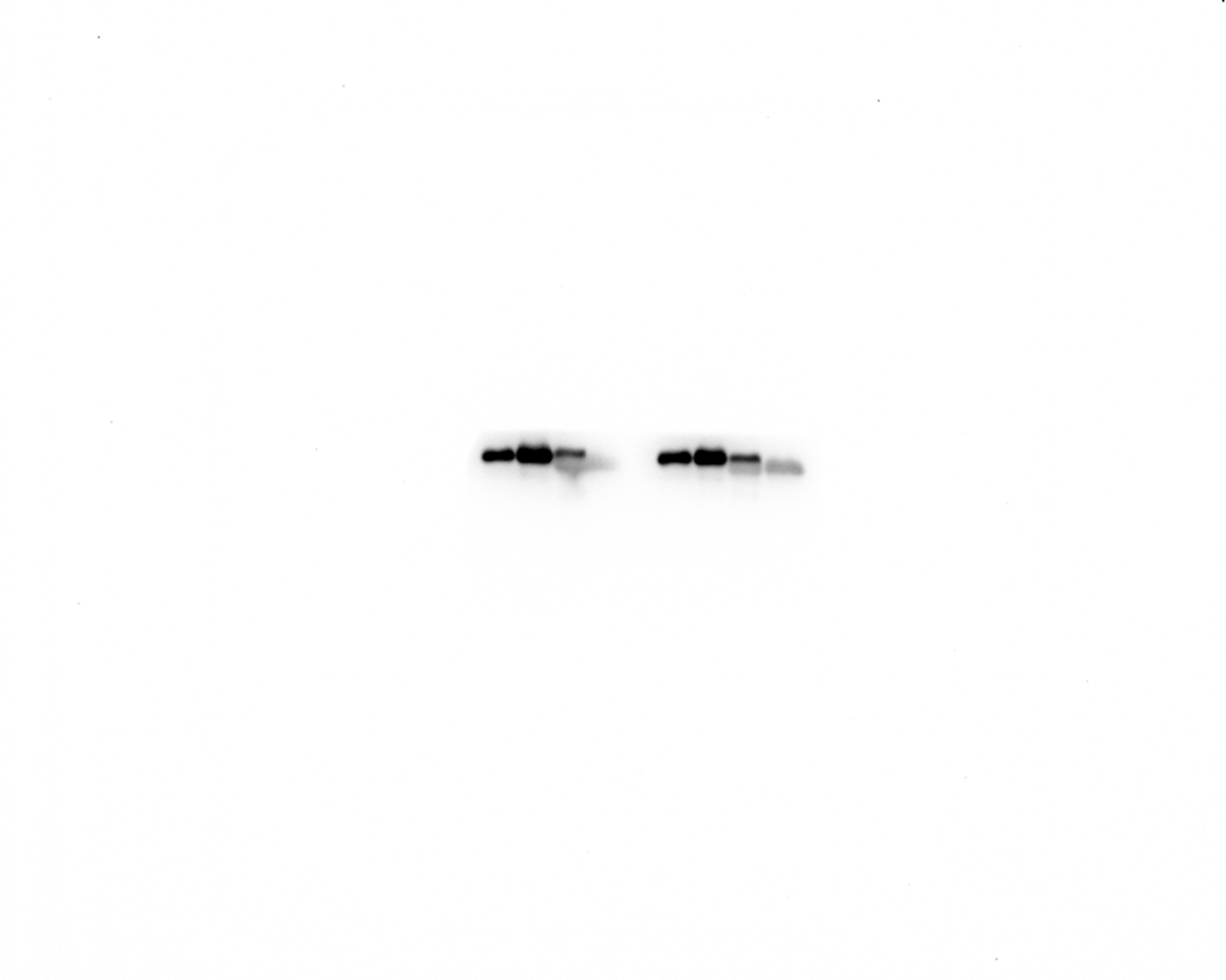

Supplement: Figure 2—source data 1. [file elife-86030-fig2-data1.zip › Figure 2G-source data/atg7-LHCIIa-CHEMI_04212022_155132_(Chemi).tif]

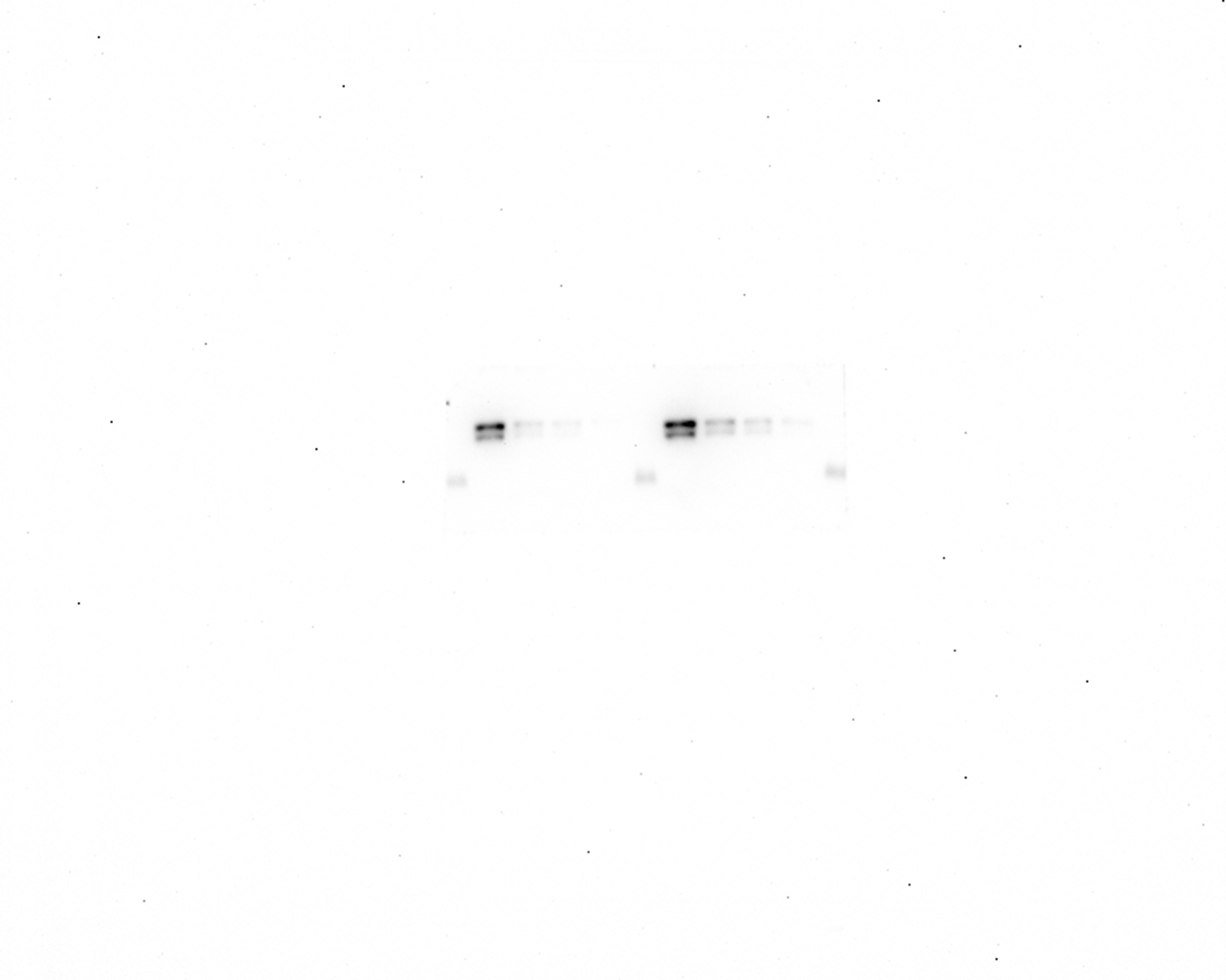

Supplement: Figure 2—source data 1. [file elife-86030-fig2-data1.zip › Figure 2G-source data/atg7-cFBPase-CHEMI_04212022_161207_(Chemi).tif]

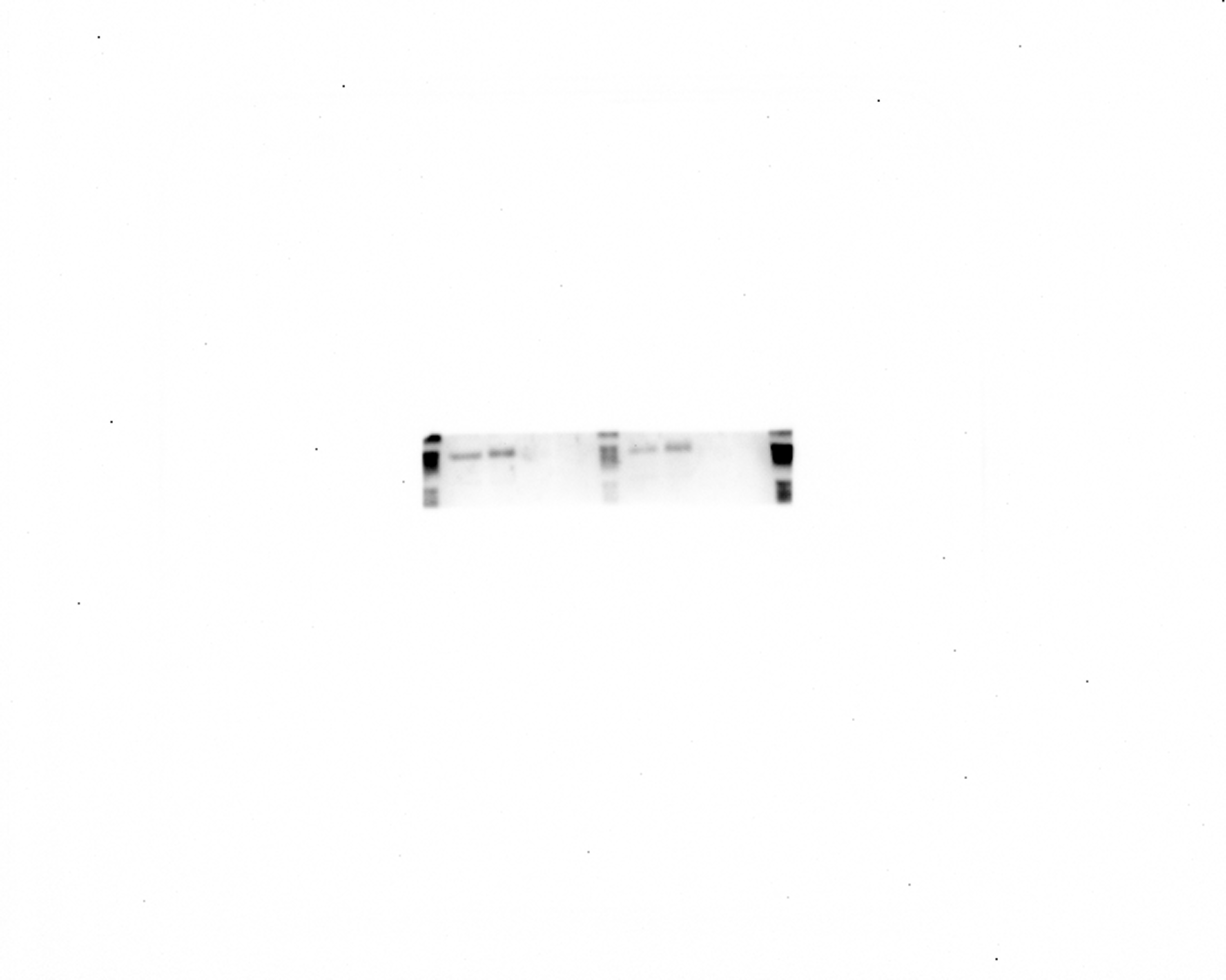

Supplement: Figure 2—source data 1. [file elife-86030-fig2-data1.zip › Figure 2G-source data/WT-TIC40-CHEMI_04212022_161454_(Chemi).tif]

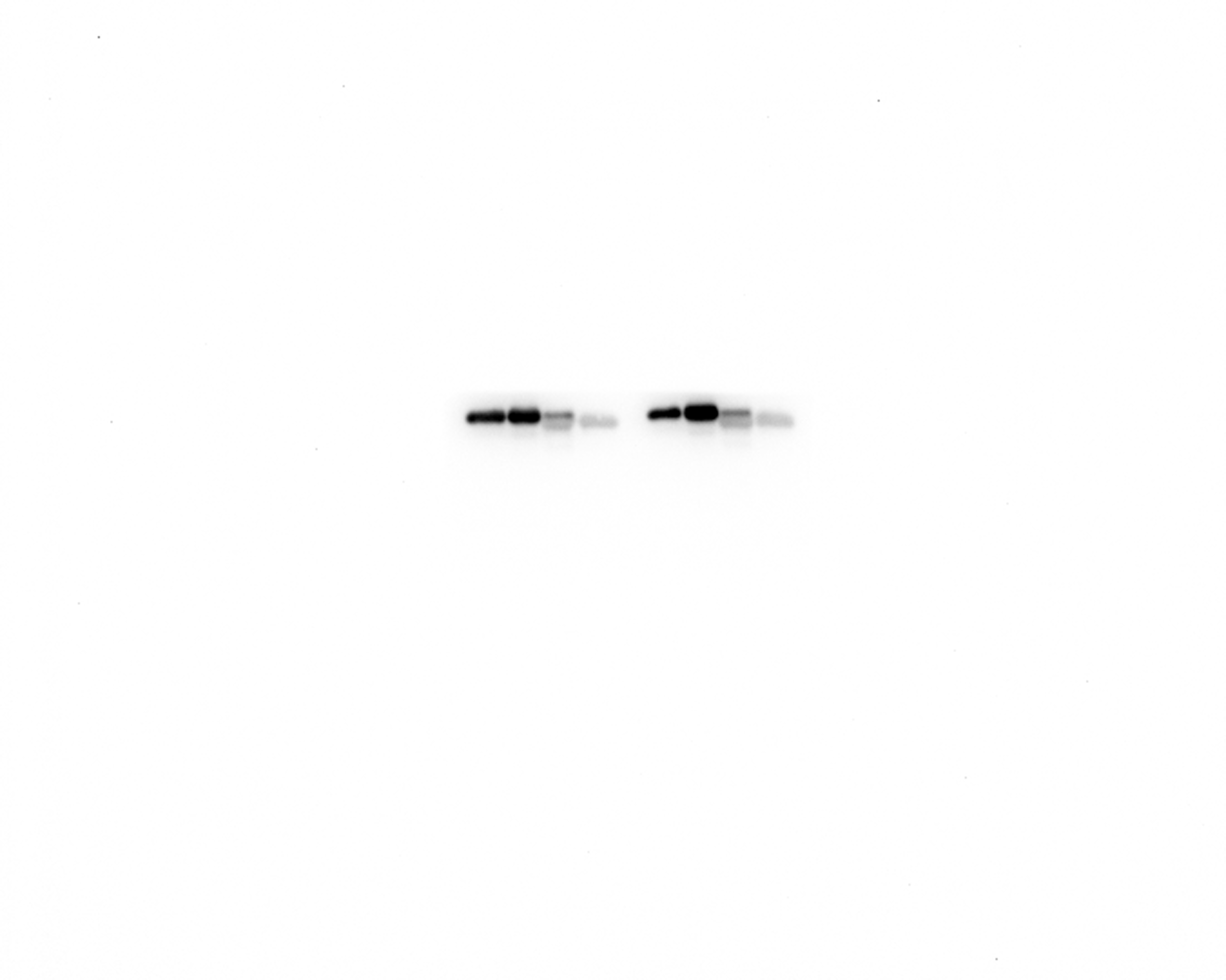

Supplement: Figure 2—source data 1. [file elife-86030-fig2-data1.zip › Figure 2G-source data/WT-LHCIIa-CHEMI_04212022_154539_(Chemi).tif]

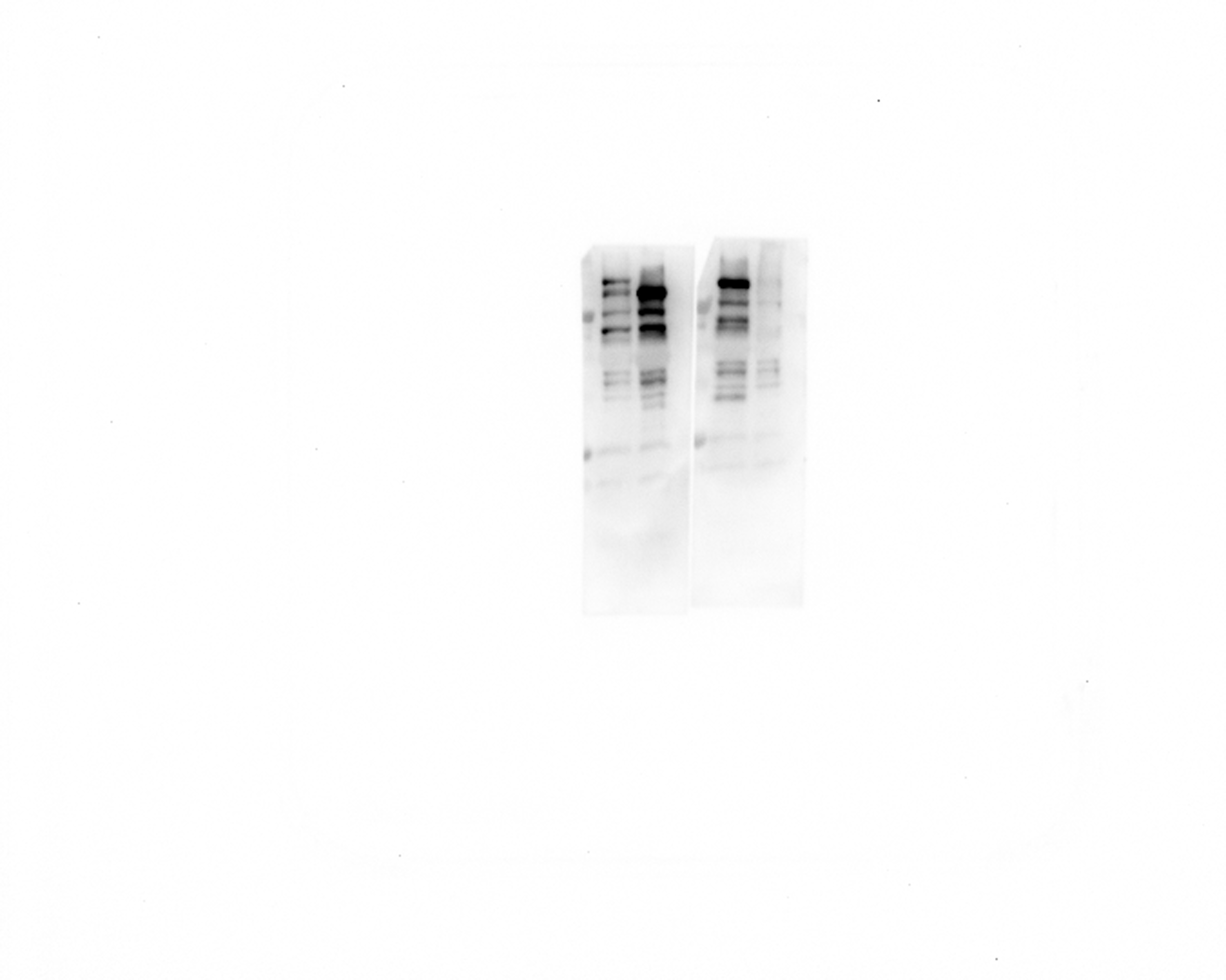

Supplement: Figure 2—figure supplement 1—source data 1. — Uncropped blots are shown in Figure 2—figure supplement 1. [file elife-86030-fig2-figsupp1-data1.zip › Figure 2-figure supplement 1-source data/anti-NBR1-CHEMI_05242022_111608_(Chemi).tif]
